# Scorpion Venom Heat-Resistant Peptide (SVHRP) Enhances Neurogenesis and Neurite Outgrowth of Immature Neurons in Adult Mice by Up-Regulating Brain-Derived Neurotrophic Factor (BDNF)

**DOI:** 10.1371/journal.pone.0109977

**Published:** 2014-10-09

**Authors:** Tao Wang, Shi-Wei Wang, Yue Zhang, Xue-Fei Wu, Yan Peng, Zhen Cao, Bi-Ying Ge, Xi Wang, Qiong Wu, Jin-Tao Lin, Wan-Qin Zhang, Shao Li, Jie Zhao

**Affiliations:** 1 Department of Physiology, Dalian Medical University, Dalian, China; 2 Liaoning Engineering Technology Centre of Target-based Nature Products for Prevention and Treatment of Ageing-related Neurodegeneration, Dalian, China; 3 The Menzies Research Institute Tasmania, University of Tasmania, Hobart, Australia; 4 Department of Biotechnology, Dalian Medical University, Dalian, China; Federal University of Rio de Janeiro, Brazil

## Abstract

Scorpion venom heat-resistant peptide (SVHRP) is a component purified from *Buthus martensii* Karsch scorpion venom. Although scorpions and their venom have been used in Traditional Chinese Medicine (TCM) to treat chronic neurological disorders, the underlying mechanisms of these treatments remain unknown. We applied SVHRP *in vitro* and *in vivo* to understand its effects on the neurogenesis and maturation of adult immature neurons and explore associated molecular mechanisms. SVHRP administration increased the number of 5-bromo-2’-dexoxyuridine (BrdU)-positive cells, BrdU- positive/neuron-specific nuclear protein (NeuN)-positive neurons, and polysialylated-neural cell adhesion molecule (PSA-NCAM)-positive immature neurons in the subventricular zone (SVZ) and subgranular zone (SGZ) of hippocampus. Furthermore immature neurons incubated with SVHRP-pretreated astrocyte-conditioned medium exhibited significantly increased neurite length compared with those incubated with normal astrocyte-conditioned medium. This neurotrophic effect was further confirmed *in vivo* by detecting an increased average single area and whole area of immature neurons in the SGZ, SVZ and olfactory bulb (OB) in the adult mouse brain. In contrast to normal astrocyte-conditioned medium, higher concentrations of brain-derived neurotrophic factor (BDNF) but not nerve growth factor (NGF) or glial cell line-derived neurotrophic factor (GDNF) was detected in the conditioned medium of SVHRP-pretreated astrocytes, and blocking BDNF using anti-BDNF antibodies eliminated these SVHRP-dependent neurotrophic effects. In SVHRP treated mouse brain, more glial fibrillary acidic protein (GFAP)-positive cells were detected. Furthermore, immunohistochemistry revealed increased numbers of GFAP/BDNF double-positive cells, which agrees with the observed changes in the culture system. This paper describes novel effects of scorpion venom-originated peptide on the stem cells and suggests the potential therapeutic values of SVHRP.

## Introduction

The scorpion *Buthus martensii* Karsch (BmK) and its venom have long been used in traditional Chinese medicine as drugs for treating chronic neurological diseases, such as epilepsy and cerebral infarction [1]. In the *Chinese Pharmacopoeia Compendium of Materia Medica*, published in the mid-16th century, the pharmacological properties and applications of the scorpion were described in detail. It is believed that scorpions and their venom are the effective components. To date, more than 400 different peptides, toxins or homologues have been purified and functionally characterized from scorpion venom, most belonging to the Na^+^ or K^+^ channel toxin families [2], [3]. Scorpion venom- originated peptides exhibit multiple pharmaceutical effects in various physiological and pathological conditions, such as anti-tumor effects [4], analgesic effects [5] and bradykinin-potentiating, antimicrobial, hemolytic and immune-modulating activities [6].

Our previous work identified one scorpion venom heat-resistant peptide (SVHRP) from BmK venom [7], this peptide exhibits protective effects by inhibiting the excitability of freshly isolated hippocampal neurons [8], [9].

Neurogenesis occurs throughout the life of mammals. In addition to the developmental stage, there are active neurogenic activities in two specific areas in the adult brain: the subventricular zone (SVZ) and subgranular zone (SGZ) of the dentate gyrus in the hippocampus [10], [11]. Neural stem cells (NSCs) or progenitors in these two areas undergo self-renewal and eventually differentiate into mature neurons and glial cells under physiological conditions. This action also occurs under certain pathological conditions, such as ischemia [12] and seizure [13]. Because suitable neural cell types are required to repair injured areas [14], [15], promoting NSCs proliferation and differentiation towards mature neurons is thus one new strategy for therapy for CNS diseases [16], [17].

It is unknown whether scorpion venom affects the functional state of stem cells. A peptide from scorpion venom has been reported to promote proliferation of mouse bone marrow cells [18]. Ts15, a peptide purified from the venom of the Brazilian scorpion, preferentially blocks Kv1.2 and Kv1.3 channels [19], whereas the blocking of Kv1.3 channels increases neural progenitor cell proliferation [20].

To determine whether peptides form scorpion venom affects the functional states of neuronal stem cells, the present study explored the effects of SVHRP on the neurogenesis and maturation of adult neural stem/progenitor cells in the SVZ and SGZ. We found that SVHRP enhances neurogenesis and stimulates neural maturation. These findings thus suggest novel functional characteristics of SVHRP and indicate its potential therapeutic value.

## Materials and Methods

### Animals

A total of 28 healthy, 3-month-old, weighing (30±3) g, C57BL/6 male mice, provided by the Animal Experimental Center of Dalian Medical University, were used for this experiment. Animals were randomly assigned to control group and SVHRP-treated group (SVHRP group). Animals were handled with the guidelines of the Committees of Animal Use and Protection and the animal studies committees of the Dalian Medical University approved these animal protocols (Ethics committee approval permit NO. L2013011).

### BrdU and SVHRP Application

To assess the newborn cells in the adult mice, BrdU (5-bromo-2’-dexoxyuridine) was injected [21]. In Brief, intraperitoneal injection of BrdU (dissolved in 0.9% NaCl, pH = 7.4; 100 mg/kg body mass) was given (6 times and once daily for 6 days) to label the slow dividing, quiescent stem cells, mice were then kept for 4 weeks before perfusion.

SVHRP isolated from BmK venom, as described [8]–[9], was freshly prepared as a stock solution (10 µg/µl) in sterile deionized and distilled water and diluted to the desired final concentrations in the treatment medium. SVHRP was diluted with saline into a final concentration of 4 µg/mL. The SVHRP-treated mice were intraperitoneally injected with 20 µg/kg body mass, while the control group received 5 ml/kg body mass saline, once per day. SVHRP was used for 4 days prior to the BrdU application, during 6 days of BrdU injection, it was used simultaneously, thus was used for a total of 10 days [22].

### Primary antibodies were the following

Glial Fibrillary Acidic Protein (GFAP) (1∶800; DAKO, Glostrup, Denmark), 5-bromo-2-deoxyuridine (BrdU) (1∶100; AB-tech, Sheffield, UK), polysialylated neural cell adhesion molecule (PSA-NCAM) (1∶800; Millipore, Billerica, MA, USA), BDNF (1∶800; Abcam, Cambridge, MA, USA),β-tubullin III (1∶800; Millipore, Billerica, MA, USA).

### Immunofluorescence

Mice were anesthetized and perfused with phosphate buffered saline (PBS) followed by 4% paraformaldehyde in phosphate buffer. The brain tissues were post-fixed in 4% paraformaldehyde for 1–2 days. Sequential 15 to 30% sucrose treatment was performed for 1–2 days, brain samples were cryosectioned (16 µm thickness). Brain sections were washed in PBS for 3 times, after blocking with 5% BSA solution for 1 h, sections were incubated with primary antibody overnight at 4°C. Alexa Fluor 488 or 594 secondary antibody (Invitrogen, USA) was added for 1 h at 37°C. 4, 6-Diamidino-2-phenylindole (DAPI) was added as nuclei counterstaining dye. A Leica DM 4000B microscope or Leica TCS SP5 microscope was used to exam staining for conventional or confocal imaging, respectively.

### Counting of Immuno-Positive Cells

Brain sections from Bregma −1.7 mm to −2.18 mm (SGZ), Bregma 0.5 mm to 0.02 mm (SVZ) of the mouse brain stereotaxic coordinates were used [23]. Immune-positive cells in these sections with 50 µm intervals in each animal were counted. Positive cells on all sections were counted and averaged to the number of SGZs and SVZs. A double blind fashion was used in all cells counting.

### Astrocytes Cultures and Preparation of Conditioned Medium

Mouse primary cortical mixed glia was prepared from the brains of 1-day-old C57 mice, as described by Liu et al [24]. Briefly, cortical tissues were triturated after removing the meninges and blood vessels. Cells were seeded into 24-well culture dishes with round coverslips at the bottom of the wells or flasks pre-coated with poly-D-lysine. Seeding medium contained DMEM supplemented with 10% FBS, 1 g/L glucose, 2 mM L-glutamine, 50 U/ml penicillin and 50 µg/ml streptomycin. The medium was changed every 3 days with seeding medium. On day 12, 10 mmol/L L-leucine-methyl-ester was added into the media for 1 h to kill microglials. Culture astrocytes were pretreated with SVHRP for 24 h (20/µg/ml). The treatment medium was Neurobasal, containing 1% FBS, L-Glutamine, antibiotics. The medium was then collected as conditioned medium. In the BDNF blocking experiment, anti BDNF antibodies (2ug/ml) was added to the conditioned medium in order to neutralize the BDNF.

### NPCs Differentiated in Astrocytes Conditional Medium

SGZs and SVZs were dissected [25] and cultured in DMEM/F12 (Gibco, USA), containing L-Glutamine, N2, B27, epidermal growth factor (EGF) and basal fibroblast growth factor. Neurospheres formed after 7–9 days culture, they were then digested and passaged to obtain neurospheres originating from a single primary cell. Secondary or tertiary neurospheres were used for subsequent experiments. Digested NSCs were plated in poly-L-lysine pretreated 24-well culture dishes (Nunc, Denmark) and maintained for 7 days in NB +2% B27 medium plus 1% fetal bovine serum with medium being half-exchanged every 3 days. Some of them were plated in 24-well culture dishes and maintained for 7 days in astrocytes conditioned medium with medium being half-exchanged every 3 days. Differentiated cells from NSCs were fixed with 4% paraformaldehyde (20 min), then permeabilized with 0.1% Triton-X-100 in PBS (15 min), and incubated with 5% BSA solution (60 min) at room temperature (RT). Primary antibodies were added and incubated overnight at 4°C. After three washing steps with PBS, the cells were incubated with the corresponding fluorescent secondary antibodies (RT, 2 h). Additionally, cells were stained with the nuclear dye DAPI (10 min). Cells were visualized using a fluorescence microscopy (Leica Microsystems DM4000B, Germany).

### Reversed Transcript Polymerase Chain Reaction

Total RNA of Adult SGZs, SVZs and cultured astrocytes was prepared by using the Trizol Reagent (Invitrogen, USA). The Superscript TM-III kit (Invitrogen, USA) was used for reverse-transcribed with oligo dT and 2.5 mg total RNA. Primer sequences were as follows: GAPDH: forward, 5’-CCC CCA ATG TAT CCG TTG TG-3’, and reverse, 5’-TAG CCC AGG ATG CCC TTT AGT-3’; GFAP: forward, 5’-TGG CCA CCA GTA ACA TGC AA-3’, and reverse, 5’-CAG TTG GCG GCG ATA GTC AT-3’; BDNF : forward, 5’-AGG AGC CCC ATC ACA ATC TC-3’ and reverse, 5’-GCC AAA AAG AGA CCA CAG CA-3’; GDNF: forward, 5’-ATG AAG TTA TGG GAT GTC GTG G-3’, and reverse, 5’-GCC GCT TGT TTA TCT GGT GA-3’; NGF: forward, 5’-GCC CAC TGG ACT AAA CTT CAG CC-3’ and reverse, 5’- CCG TGG CTG TGG TCT TAT CTC-3’. The resulting cDNA PCR amplification was performed by using the following protocol for 30 cycles. An initial denaturation step was performed at 94°C for 5 min, and then denaturation at 95°C for 30 sec, annealing at 65°C for 1 min, and elongation at 72°C for 30 sec. 1% ethidium bromide stained agarose gels were used to analyze PCR products. Images were captured with a Molecular Imager Chemic Doc XR system (Bio-Rad, USA).

### Western Blot

Proteins from microdissected SGZs and SVZs were extracted in RIPA buffer. After incubated for 10 min, tissue extracts were centrifuged for 25 min. Protein content was determined by using the BCA protein assay (Pierce, USA). 25–50 µg of proteins per lane were loaded for SDS-polyacryl-amide gel electrophoresis equally and then transferred to an Amersham Hybond-P membrane (Millipore, USA). After blocking with 5% non-fat milk in for 1 h at room temperature, the membranes were incubated with primary antibodies at 4°C overnight and washed in Tris-buffered saline containing 0.1% Tween 20 (TBS-T) for 4 times. A chemiluminescence detection system (ECL, Amersham, England) was used to detect the antigen-antibody complexes. A Molecular Imager Chemic Doc XR system (Bio-Rad, Richmond, USA) was used to detect signals.

### Enzyme-Linked Immunosorbent Assays (ELISA)

Astroctyes were pretreated with SVHRP for 24 h before supernatants were collected. The levels of BDNF in the culture medium and tissue extracts were assessed with BDNF E_max_ Immuno Assay System (Promega G7610) according to the manufacturer’s instructions. A standard reference curve was prepared for each assay for accurate quantitation of BDNF levels in experimental samples.

### Data Analysis

More than three replicates were performed in each condition. All data were presented as the mean ± SEM. To make comparison between two groups, statistical analysis was carried out by *t* test. *p<0.05* was considered to be statistically significant.

## Results

### SVHRP increases cell proliferation and neurogenesis in the SGZ and OB of adult mice

**Figure 1:**
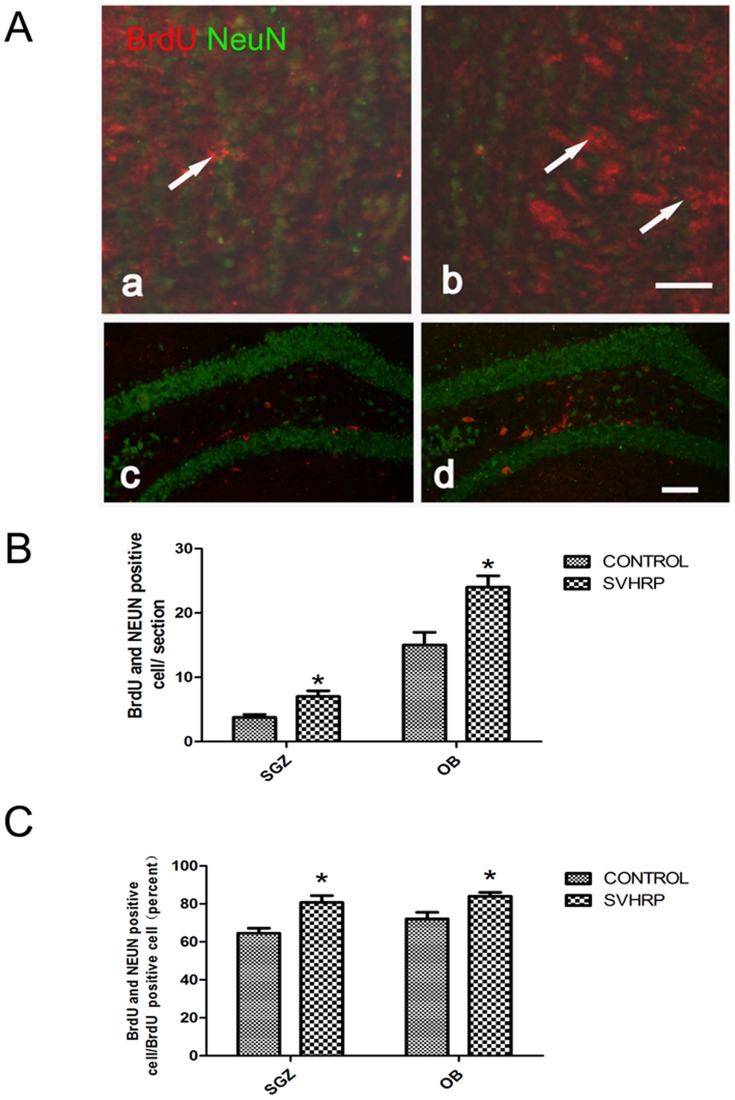
SVHRP increases the number of BrdU-retaining cells in the adult OB and SGZ. A) Long-term BrdU-retaining cells in the adult OB and SGZ. Immunostaining of long-term-retained BrdU (red) merged with NeuN (green) in Control (a.c) and SVHRP group (b.d). Scale bars represent 100 µm. B) The quantification of long-term BrdU/NeuN positive cells per OB and SGZ slice. The bar graph shows cell numbers of BrdU/NeuN positive cells (mean ± SEM) in OB and SGZ section. Control group *vs* SVHRP group; (*p<0.05, n = 4). C) The quantification of long-term BrdU/NeuN-positive cells/BrdU-positive cells in OB and SGZ slices. The bar graph shows the BrdU/NeuN-positive cells/BrdU-positive cells (mean ± SEM) in the OB and SGZ sections. Control group *vs* SVHRP group, *p<0.05, n = 4.

Neurogenesis in hippocampal SGZ and OB was labeled by BrdU/NeuN staining. Four weeks after SVHRP treatment, an increase in newly generated cells (BrdU-positive) was observed in the SGZ and OB in SVHRP-treated mice compared with control mice (Fig. 1A, B, C). Some cells were NeuN-positive, indicating that newly generated cells had previously matured into neurons (BrdU and NeuN double-positive) in both SGZ and OB. Increased numbers of BrdU/NeuN double-positive cells were observed after SVHRP administration (*p<0.05*, Fig.1A, B). The ratio of BrdU/NeuN double-positive cells to the total BrdU positive-cells was also increased (*p<0.05*, Fig. 1A, C).

**Figure 2:**
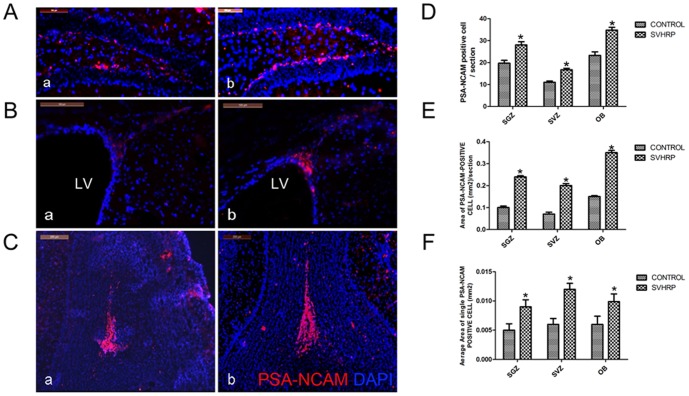
SVHRP increases the number and areas of PSA-NCAM-positive cells in the adult SGZ, SVZ and OB. Immunostaining of PSA-NCAM (red) merged with DAPI (blue). Scale bars represent 100 µm. a. Control; b. SVHRP. A) in SGZ. B) in SVZ. LV: lateral ventricle. C). in OB. Scale bars represent 200 µm. The quantification of PSA-NCAM positive cells per SGZ, SVZ and OB slice. Control group *vs* SVHRP group. D) PSA-NCAM-positive cells number. The bar graph represents cell numbers of PSA-NCAM-positive cells (mean ± SEM) in SGZ, SVZ, OB sections (*p<0.05, n = 5). E) The areas of PSA-NCAM-positive cells. The bar graph represents areas of PSA-NCAM positive cells (mean ± SEM) in SGZ, SVZ, OB sections (*p<0.05, n = 5). F) The average area of single PSA-NCAM-positive cells. The bar graph shows the average area of single PSA-NCAM-positive cells (mean ± SEM) in SGZ, SVZ and OB sections (*p<0.05, n = 5).

Polysialylated-neural cell adhesion molecule (PSA-NCAM) is a marker of neuroblasts, which ultimately differentiate into mature neurons in the OB and SVZ [26]–[28]. To determine whether SVHRP can also increase the number of immature neurons, PSA-NCAM immunofluorescence staining in the OB, SVZ and SGZ of adult mice was analyzed. These analyses showed that the number of PSA-NCAM-positive cells was significantly increased in the OB, SVZ and SGZ of SVHRP-treated mice (Fig. 2Aa, Ba, Ca, D) compared with untreated adult mice (Fig. 2Ab, Bb, Cb, D) (*p<0.05*). The whole area and the average single area of immature neurons in the normal adult SGZ, SVZ and OB were also increased after SVHRP administration (Fig. 2E, F).

### SVHRP increases the expression of GFAP and Nestin double-positive cells in the adult SGZ and SVZ

**Figure 3:**
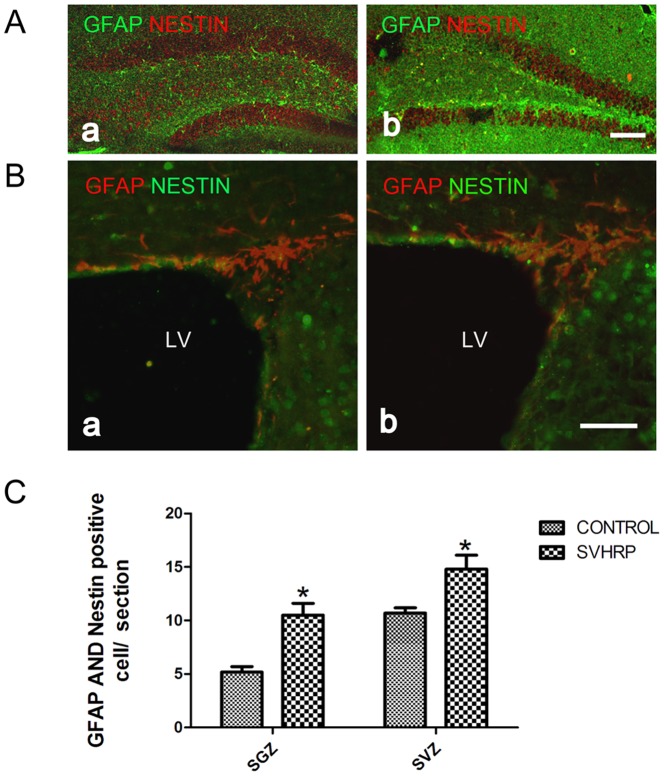
SVHRP increases the number of GFAP/Nestin-positive cells in the adult SGZ and SVZ. A) Immunostaining of GFAP (green) and Nestin (red) in the SGZ. a. Control; b. SVHRP. B) Immunostaining of GFAP (red) and Nestin (green) in the SVZ. a. Control; b. SVHRP. LV: lateral ventricle. C) The quantification of GFAP- and Nestin-positive cells per SGZ and SVZ section. The bar graph shows the numbers of GFAP- and Nestin-positive cells (mean ± SEM) in SGZ and SVZ section (*p<0.05, n = 3).

The neuroblasts in the SGZ and SVZ originate from non-radial type 2 cells and C cells, which are generated from the type 1 progenitor cells and B cells, respectively. These stem/progenitor cells exhibit a radial glia-like appearance and are GFAP^+/^Nestin^+^-positive [29]. To study the effects of SVHRP on type 1 precursor cells and B cells, GFAP and Nestin double immunofluorescence staining was performed. We found that the number of GFAP^+^/Nestin^+^ radial glia-like precursors in the SGZ and SVZ was significantly greater in SVHRP-treated mice than that of controls (*p<0.05*, Fig. 3A, B, C).

### SVHRP increases the length of neurites in astrocyte-conditioned medium

**Figure 4:**
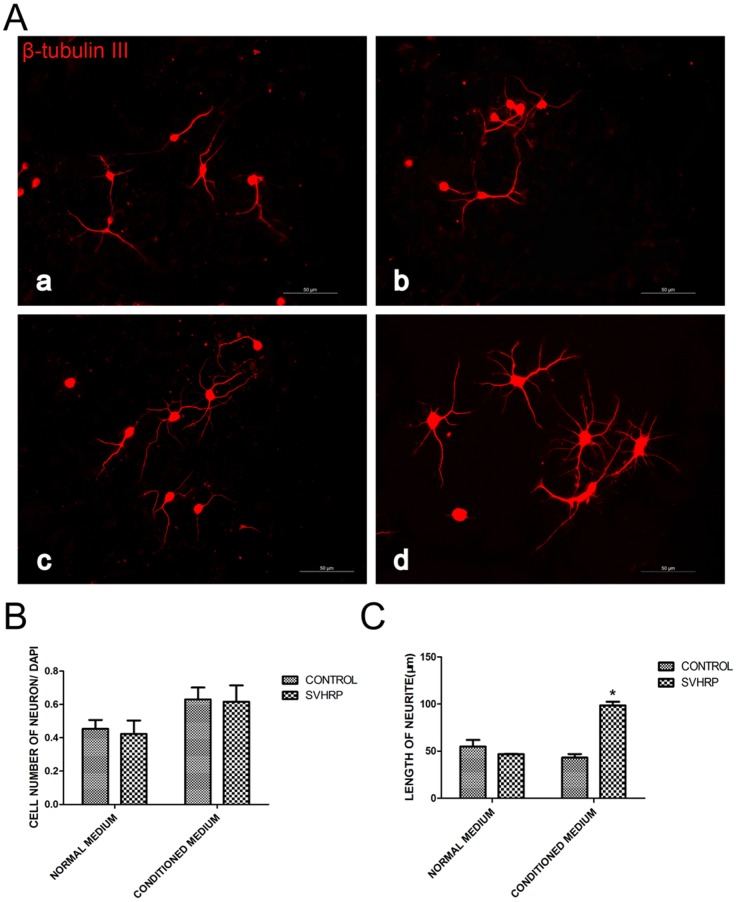
SVHRP-pretreated conditioned medium increases the length of neuronal neurites. A) Immunostaining of β-tubulin III (red) in immature neurons differentiated form NPCs. Scale bars represent 50 µm. a. Control normal medium; b. SVHRP-treated normal medium; c. Control conditioned medium; d. SVHRP-treated conditioned medium. B) Quantification of the proportion of neurons (neurons/DAPI). The bar graph shows the proportion of neurons (neurons/DAPI), n = 4. C) Quantification of length of neuron neurites. The bar graph shows the length of neuron neurites (mean ± SEM). Control group *vs* SVHRP group in conditioned medium (*p<0.05, n = 4).

We found that the whole area and average single area of immature neurons (PSA-NCAM positive-cell) in normal adult SGZ, SVZ and OB were increased in SVHRP-treated mice compared with control mice (Fig. 2E, F), indicating a potential neurotrophic effect of SVHRP. To explore the mechanism of this effect, NPC differentiation was analyzed. Although incubation with normal astrocyte-conditioned medium resulted in no difference in neurite length of β-tubulin III-positive neurons. SVHRP-treated astrocyte-conditioned medium significantly increased the neurite length of β-tubulin III-positive neurons (*p*<0.05, Fig. 4A, B, C).

### SVHRP increases BDNF expression in cultured SVHRP-pretreated astrocytes

**Figure 5:**
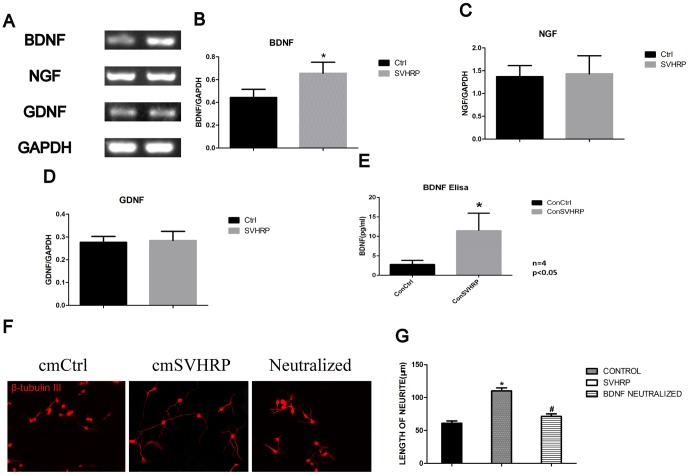
SVHRP increases BDNF expression in cultured astrocytes. A) RT-PCR of BDNF, NGF, GDNF and GAPDH in primary astrocyte cells culture. B) Quantification of BDNF mRNA in primary astrocyte cells. The bar graph shows the intensity of BDNF/GAPDH (mean ± SEM) in primary astrocytes cell culture. Control group *vs* SVHRP group, *p<0.05, n = 4. C) Quantification NGF mRNA in primary astrocyte cell culture. The bar graph shows the intensity of NGF/GAPDH (mean ± SEM) in primary astrocytes. Control group *vs* SVHRP group, n = 4. D) Quantification GDNF mRNA in primary astrocytes. The bar graph shows the intensity of GDNF/GAPDH (mean ± SEM) in primary astrocytes. Control group *vs* SVHRP group, n = 4. E) Primary astrocytes were pretreated with SVHRP (20 µg/ml), and the amount of BDNF in the supernatant was measured with ELISA assays. The bar graph shows the amount of BDNF (mean ± SEM). Control group *vs* SVHRP group (*p<0.05, n = 3). F) Immunostaining of β-tubulin III (red) in immature neurons differentiated form NPCs. Scale bars represent 50 µm. a. Control conditioned medium; b. SVHRP-treated conditioned medium. c. BNDF-neutralized SVHRP-treated conditioned medium. G) Quantification of neurites length of. The bar graph shows length of neuron neurites (mean ± SEM). Control group *vs* SVHRP group in conditioned medium (*p<0.05, n = 3), and SVHRP group *vs* BNDF-neutralized SVHRP group in conditioned medium (*#p<0.05*, n = 3).

NGF, GDNF and BDNF are the main growth factors secreted by astrocytes, which are important for the proliferation and maturation of stem cells. To identify the growth factors that participate in SVHRP’s neurotrophic effects, the mRNA expression levels of NGF, GDNF and BDNF were analyzed. No differences in NGF and GDNF mRNA levels in SVHRP-treated astrocytes were observed compared with normal astrocytes (Fig. 5A, C, D). mRNA level of BDNF in SVHRP-treated astrocytes were increased compared with normal astrocytes (*p*<0.05, Fig. 5A, B). Secreted BDNF in the SVHRP-treated astrocytes culture medium was also increased, which was confirmed by ELISA assay (*p*<0.05, Fig. 5E). To determine whether the effects of SVHRP on neurite outgrowth were principally due to BDNF, we inhibited BDNF in SVHRP-treated astrocyte-conditioned medium by applying anti-BDNF antibodies and performed the neurite outgrowth analysis under the BDNF blocking conditions. SVHRP administration failed to increase the length of neurites (*p*<0.05, Fig. 5F, G).

### SVHRP increases the expression of GFAP in the adult SGZ and SVZ

In addition to the direct neurogenic properties of GFAP-positive type-1 cells (SGZ) and B1 cells (SVZ), GFAP-positive astrocytes also play multiple roles in promoting the proliferation of precursor cells in neurogenic zones. For example, these cells serve as niche cells, secret extracellular matrix molecules and release paracrine factors and growth factors [30].

**Figure 6:**
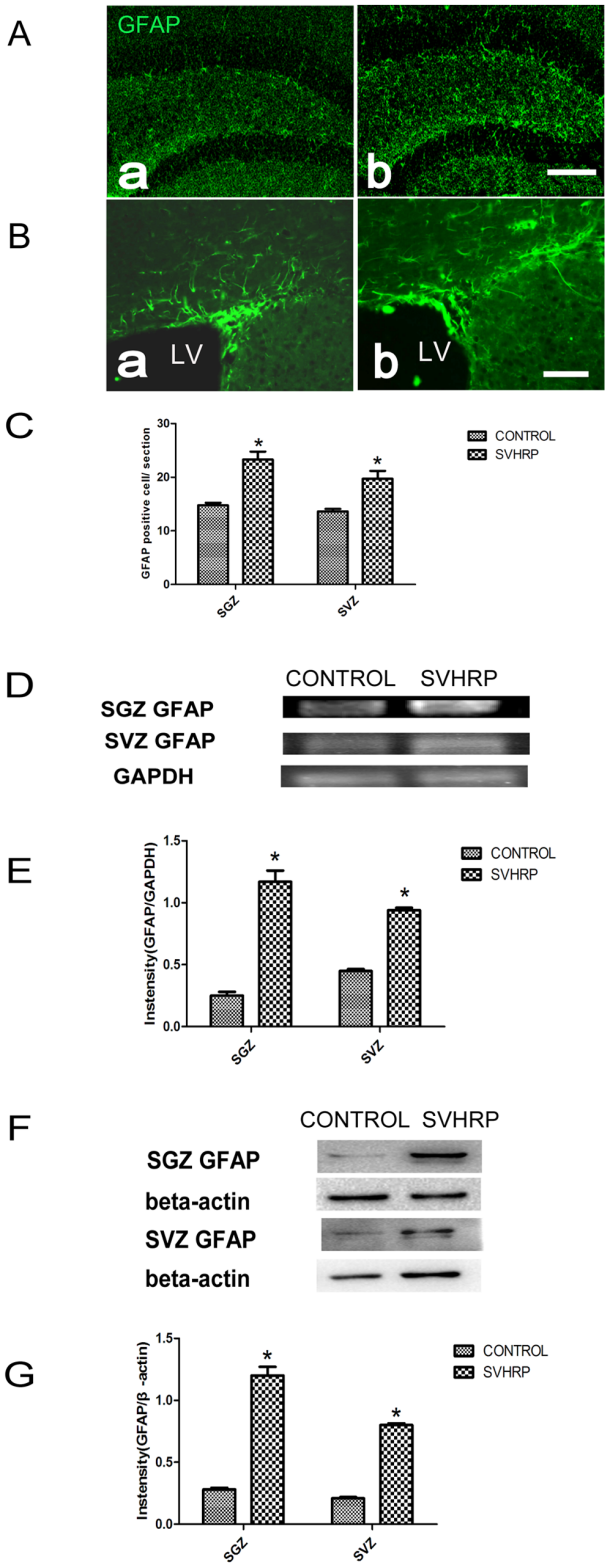
SVHRP increases the number GFAP-positive cells in the adult SGZ and SVZ. Immunostaining of GFAP (green). Scale bars represent 100 µm. a. Control; b. SVHRP. A) in SGZ. B) in SVZ. LV: lateral ventricle. C) Quantification of GFAP-positive cells per SVZ and SGZ slice. The bar graph shows numbers of GFAP-positive cells (mean ± SEM) in SVZ and SGZ. Control group *vs* SVHRP group, *p<0.05, n = 4. D) RT-PCR of GFAP in the SVZ and SGZ. E) Quantification of GFAP mRNA levels in the SVZ and SGZ. The bar graph shows the intensity of GFAP/GAPDH (mean ± SEM) in the SVZ and SGZ section. Control group *vs* SVHRP group, *p<0.05, n = 5. F) Western Blot of GFAP in SVZ and SGZ. G) Quantification of GFAP protein levels in the SVZ and SGZ. The bar graph shows Intensity of GFAP/β-actin (mean ± SEM) in the SVZ and SGZ. Control group *vs* SVHRP group, *p<0.05, n = 4.

To elucidate the effect of SVHRP on GFAP-positive astrocytes in the hippocampus, the number of GFAP-positive cells, as well as the mRNA and protein expression levels of GFAP were analyzed. More GFAP-positive cells were observed in the SGZ and SVZ of SVHRP-treated mice (*p<0.05*, Fig. 6Ab, Bb, C) compared with control group (Fig. 6Aa, Ba, C). In addition, mRNA (Fig. 6D, F) and protein (Fig. 6E, G) expression levels of GFAP were increased after SVHRP administration (*p<0.05*).

### SVHRP increases the expression of BDNF in astrocytes of the adult SGZ and SVZ

**Figure 7:**
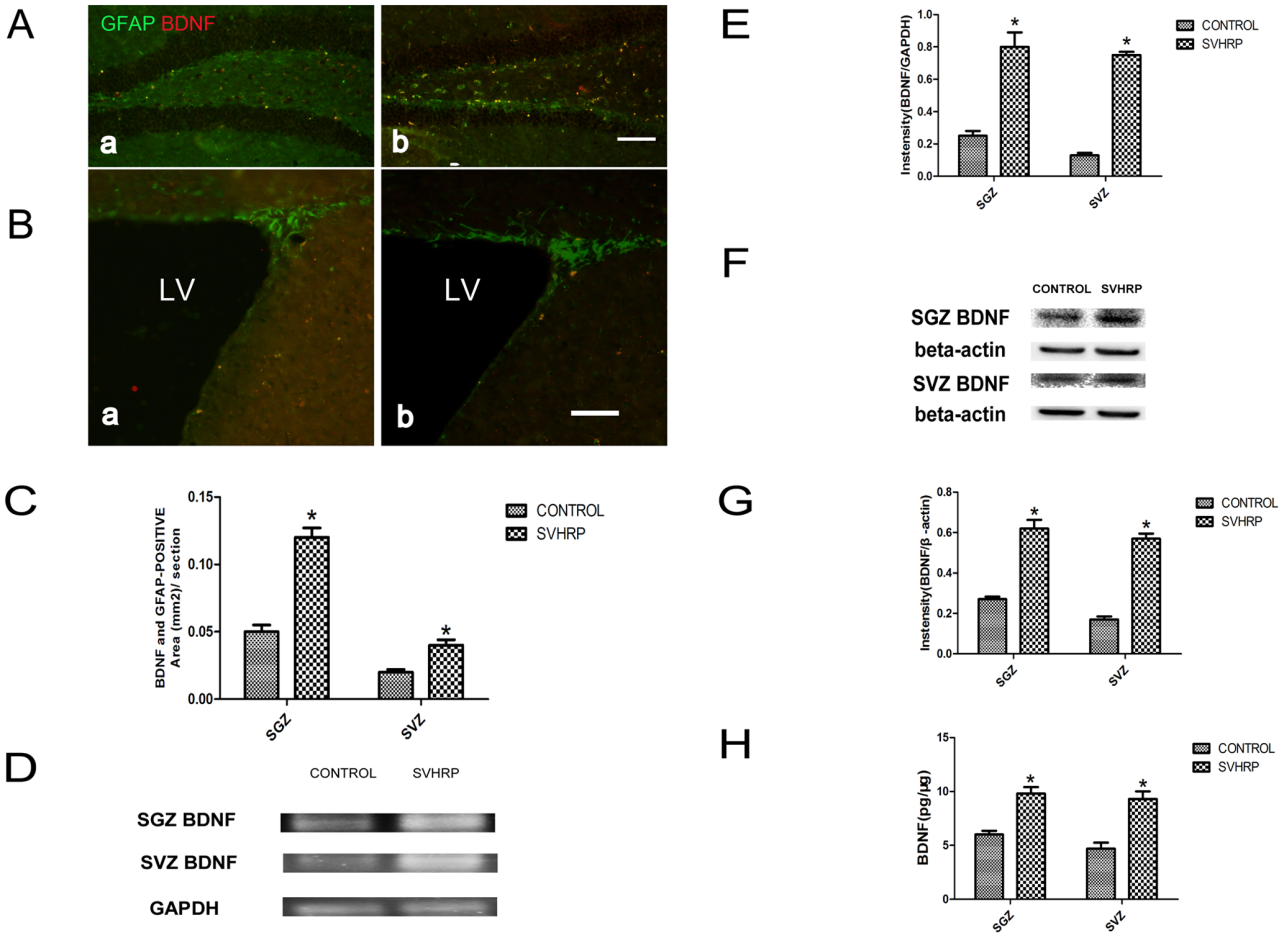
SVHRP increases BDNF expression in the adult SGZ and SVZ. Immunostaining of BDNF (red) and GFAP (green). Scale bars represent 100 µm. a. Control; b. SVHRP. A) in SGZ. B) in SVZ. LV: lateral ventricle. C) Quantification of BDNF- and GFAP- positive cells per SGZ and SVZ slice. The bar graph shows the area of BDNF- and GFAP-positive cells (mean ± SEM) in SGZ and SVZ sections. Control group *vs* SVHRP group, *p<0.05, n = 3. D) RT-PCR of BDNF in the SVZ and SGZ. E) Quantification BDNF mRNA levels in the SVZ and SGZ. The bar graph shows the intensity of GFAP/GAPDH (mean ± SEM) in SVZ and SGZ sections. Control group *vs* SVHRP group, *p<0.05, n = 4. F) BDNF protein levels in the SVZ and SGZ. G) Quantification of BDNF protein levels of in SVZ and SGZ. The bar graph shows the intensity of BDNF/β-actin (mean ± SEM) in SVZ and SGZ. Control group *vs* SVHRP group *p<0.05, n = 3. H) BDNF in the in SVZ and SGZ was measured with ELISA assay. The bar graph represents amount of BDNF (mean ± SEM) in SVZ and SGZ. Control group *vs* SVHRP group *p<0.05, n = 4.

To determine whether the same dynamic change in BDNF also occurs in vivo, GFAP and BDNF double staining was analyzed in both the SGZ and SVZ. We observed more positive signals in SVHRP-treated mouse brains (*p*<0.05, Fig. 7A, B, C). mRNA and protein levels of BDNF in the adult SGZ and SVZ were elevated in the SVHRP-treated group compared with the control group (*p*<0.05, Fig. 7D, E, F, G). Secreted BDNF in the SVHRP-treated SVZ and SGZ was also increased, which was confirmed by ELISA assays (*p*<0.05, Fig. 7H). These results suggest that SVHRP increase BDNF expression in the astrocytes of the SGZ and SVZ.

## Discussion

In the present study, SVHRP increased the neurogenesis in both the SGZ and OB (BrdU/NeuN positive) (Fig. 1Aa-d, B). The proportion of BrdU-positive cells expressing NeuN was higher in SVHRP-treated adult mice than in control mice (Fig. 1C), indicating more active neurogenesis in these areas. Because mature neurons originate from neuroblasts, a group of precursor cells committed to the neuronal fate, we investigated whether enhanced neurogenesis resulted from an increase in neuroblast generation. The expression of PSA-NCAM, a marker of immature neurons, was analyzed, and a substantial increase in PSA-NCAM was identified after SVHRP treatment (Fig. 2).

GFAP^+^/Nestin^+^ radial glia-like precursors have commonly been believed to be NPCs in adult SGZ and SVZ [10], [11], [29]. They undergo slow symmetric cell divisions to maintain the neural stem cell population, However, asymmetric cell divisions result in highly proliferative daughter cells that eventually convert to neuronal progenitor cells (PSA-NCAM-positive immature neurons) [29]. The present study identified an increase in GFAP^+^/Nestin^+^ cells after SVHRP application (Fig 3), which may be the immediate cause of the increasing number of immature neurons in SVHRP-treated mice.

GFAP-expressing cells could be neuronal stem cells or postmitotic astrocytes, the latter of which also participate in neurogenesis in multiple manners. They promote the proliferation of precursor cells and their neuronal differentiation [29] by secreting extracellular matrix molecules such as thrombospondins or Netrin-4 [31.32]. Astrocytes can also release growth factors and neurotrophic factors, such as fibroblast growth factor-2 (FGF-2), which are critical for the maintenance and expression of the precursor cell pool [33]. Apart from these supporting roles in stem cell niches, astrocytes can be activated after brain injury and acquire or reactivate stem cell potential as part of reactive gliosis. This regenerative potential could be utilized for therapeutic approaches [34]. The present study identified a general increase in GFAP expression using both morphology and biochemical procedures (Fig. 6) after SVHRP treatment. This up-regulation may be one of the mechanisms underlying the increase in the number of GFAP^+^/Nestin^+^ type 1 stem cells and immature neurons (PSA-NCAM-positive).

The extrinsic local microenvironment or “niche” of NSCs, including growth factors, cytokines and cell–cell contact, plays a key role in determining the cell fate of stem cells [35]–[37]. Astrocyte-conditioned medium has been reported to have a neuro-protective effect on the survival of rat embryonic cortical neurons with secreted NT3, BDNF [38]–[39], demonstrating that astrocytes are important for the microenvironment. In the present study, we found that the length of immature neuron neurites in SVHRP-pretreated astrocytes-conditional medium was increased compared with that in normal astrocyte-conditional medium (Fig. 4). This phenotype agrees with the finding that SVHRP improved the whole area and the average single area of immature neurons in the normal adult SGZ, SVZ and OB (Fig. 2E, F). We then speculated that SVHRP may affect neuron maturation by modulating the functional states of astrocytes. Indeed, we observed elevated levels of BDNF in astrocyte-conditioned medium with SVHRP treatment (Fig 5A, B, E). To investigate whether the primitive effect of neurite outgrowth of SVHRP- treated astrocyte-conditioned medium was mediated via BDNF, we blocked the action of BDNF using anti-BDNF antibodies and repeated the neurite length experiment. The data showed that inhibiting BDNF eliminated the stimulating effect of SVHRP (Fig. 5F, G). Concomitant with the increased GFAP expression, the astrocytes secreted more BDNF in vivo after SVHRP administration. Altogether, SVHRP-treated astrocytes secreted more BDNF and altered the whole microenvironment of NSCs, which was important for the maturity of immature neurons.

In conclusion, we revealed the novel functions of scorpion venom-purified SVHRP in enhancing neurogenesis and promoting maturation of newly generated immature neurons in adult animals, suggesting the potential therapeutic value in neurogenesis/maturation-associated neurological conditions.
